# Supplementary material for: Muscle coordination retraining inspired by musculoskeletal simulations reduces knee contact force
Source: Sci Rep. 2022 Jul 7;12:9842. doi: 10.1038/s41598-022-13386-9 (PMC9262899; doi:10.1038/s41598-022-13386-9)
Supplement: Supplementary file 1 — Supplementary Information. [file 41598_2022_13386_MOESM1_ESM.pdf]

Supplementary Information

Article: Muscle coordination retraining inspired by musculoskeletal simulations reduces knee contact force

Scott D. Uhlrich, Rachel W. Jackson, Ajay Seth, Julie A. Kolesar, Scott L. Delp

Scientific Reports

Table of Contents

Effect of gastrocnemius avoidance gait on medio-lateral knee loading metrics 3
Effect of gastrocnemius avoidance gait on hip and ankle contact force 6
Effect of gastrocnemius avoidance gait on the contralateral limb 8
Explanation of biofeedback given to participants during the experiment 11
Hip abductor muscle path adjustments 13
Passive muscle force calibration 15
Tendon compliance sensitivity analysis 19

Effect of gastrocnemius avoidance gait on medio-lateral knee loading metrics

The muscle coordination retraining intervention aimed to reduce the second peak of total knee contact force. However, it is important to evaluate the effect of this modification on other measures of knee loading. We evaluated the effect of gastrocnemius avoidance gait on medial and lateral knee contact force. We also evaluated the external knee adduction moment, an estimate of the medio-lateral distribution of knee contact force that is commonly used in the medial knee osteoarthritis literature. Compared to estimates of joint contact force from a musculoskeletal simulation, the clinical relevance of the knee adduction moment is more established[1–4], which may be related to its ease of computation. Further work is needed in the field to identify the most salient loading targets for non-surgical interventions.

We computed the medial and lateral knee contact forces from the single degree-of-freedom knee using a moment balance equation, and we determined the intercondylar distance using a regression equation based on medial and lateral knee marker positions[5]. We computed the knee adduction moment using joint reaction force analysis from a simulation that was actuated with ideal torque actuators (i.e., muscle forces did not contribute to the joint reaction analysis), and it is reported as an external moment expressed in the proximal tibial reference frame. Contact forces were normalized by bodyweight (BW), moments were normalized by BW and height (ht), and peak values were extracted from the first and second 50% of the stance phase for the baseline and retention trials. We report values for the eight subjects who retained a reduction in gastrocnemius activity during the retention trial. After ensuring normality using a Shapiro Wilk test, two-sided, paired t-tests were used to compare peaks between trials. Measures that were not normally distributed are compared with a Wilcoxon signed rank test. Due to the exploratory nature of this analysis, p-values were not corrected for multiple comparisons. All values are reported as mean ± standard deviation, and $\alpha=0.05$.

When walking with a gastrocnemius avoidance gait pattern, the first peak of the knee adduction moment increased by 0.21±0.14 %BW*ht (6.2±4.3%, $p=0.004$, t-test, $n=8$) compared to

the baseline trial, but the second peak did not change significantly (0.06±0.26 %BW*ht, 2.3±9.8%, p=0.513, t-test, n=8, Fig. S1). The first peak of medial knee contact force did not change significantly between the baseline and retention trials (0.06±0.20 BW, 3.1±9.6%, p=0.383, Wilcoxon signed rank test, n=8), but the second peak of medial contact force decreased during the retention trial (-0.34±0.33, -15±13%, p=0.024, t-test, n=8). The first peak of lateral contact force increased during the retention trial compared to baseline (0.11±0.11 BW, 11±11%, p=0.020, t-test, n=8), but the second peak was not significantly different between trials (-0.04±0.13 BW, -4.3±11%, p=0.405, t-test, n=8).

The gastrocnemius avoidance gait pattern reduced the second peak of both total and medial knee contact force but did not significantly reduce the second peak of lateral contact force. This could be due to greater reductions in gastrocnemius medialis activity compared to gastrocnemius laterals activity (Fig. 5). Only the gastrocnemius medialis activity was provided as real-time feedback to participants, so future studies could provide feedback on both heads of the gastrocnemius or the lateral head alone, depending on which compartment of the knee they are aiming to offload. Interestingly, the intervention increased the first peak of knee adduction moment but did not significantly change the first peak of medial contact force; the intervention did not significantly change the second peak of the knee adduction moment but reduced the second peak of medial contact force. These inconsistencies highlight how changes in the knee adduction moment can fail to represent changes in medial compartment loading[6].

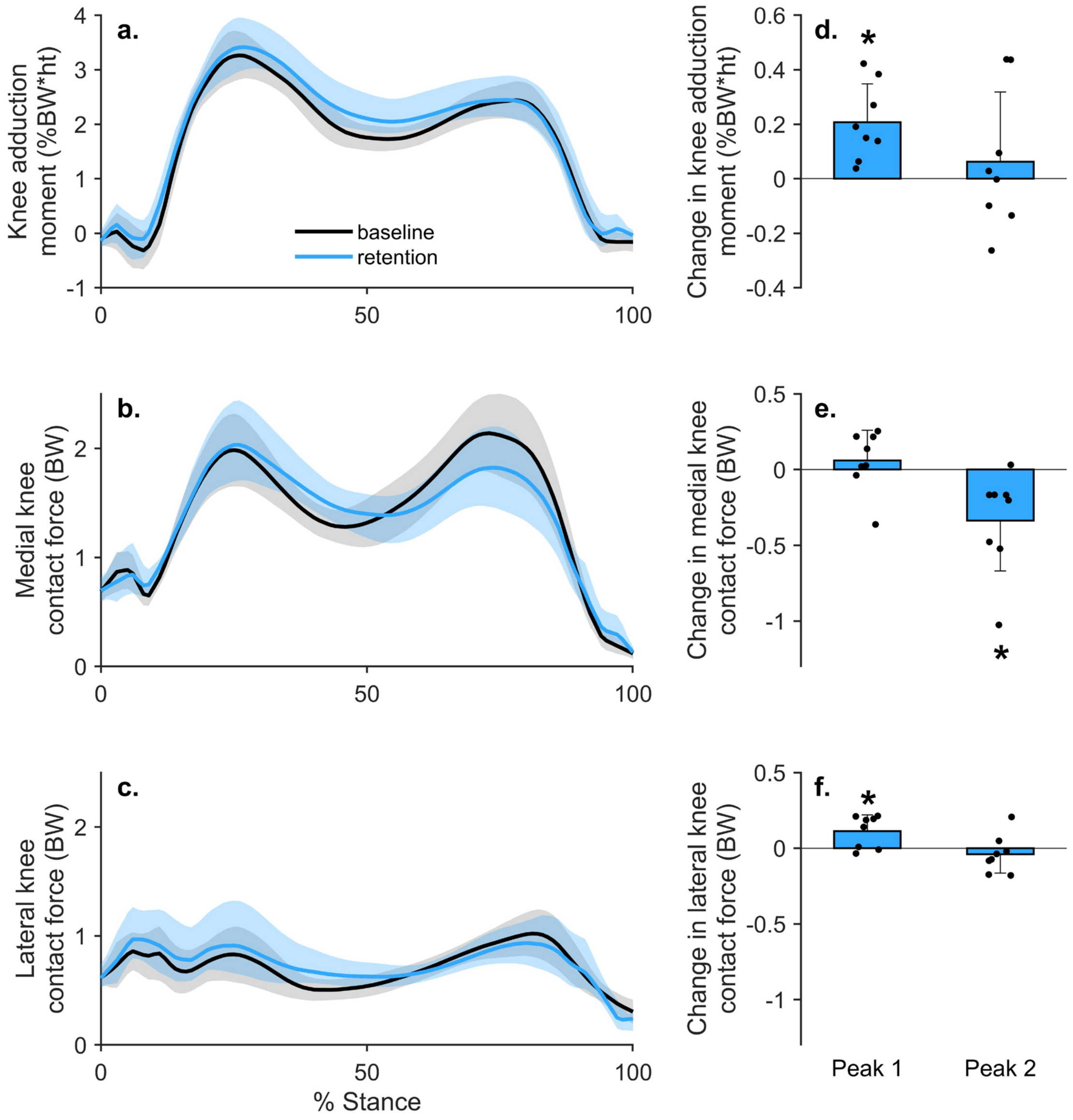

Figure S1: The effect of a gastrocnemius avoidance gait pattern on measures of medial and lateral knee loading. (a-c) The mean (line) and standard deviation (shading) of the external knee adduction moment (normalized by bodyweight [BW] and height [ht]), medial knee contact force, and lateral knee contact force for the baseline and retention trials for the eight individuals who walked with reduced gastrocnemius activation during the retention trial. **(d-e)** The mean (bar), standard deviation (error bar), and individual changes (dots) in loading measures from the baseline to the retention trial (*p<0.05). When walking with a gastrocnemius avoidance gait, the first peak of the knee adduction moment increased by 6.2±4.3% (p=0.004, paired t-test, n=8), the second peak of medial contact force decreased by 15±13% (p=0.024, paired t-test, n=8), and the first peak of lateral contact force increased by 11±11% (p=0.020, paired t-test, n=8).

Effect of gastrocnemius avoidance gait on hip and ankle contact force

We evaluated the effect of the gastrocnemius avoidance gait pattern on hip and ankle joint contact forces. The gastrocnemius avoidance gait pattern reduced the second peak of knee contact force (Fig. 6); however, the compensatory muscle force that could be used to walk with identical kinetics but reduce gastrocnemius activation could increase joint contact forces at other lower extremity joints, especially the hip (Fig. 3). We evaluated contact forces from the same simulated gait cycles as were used to evaluate knee contact force. Hip contact force was defined as the force acting on the femur from the pelvis along the long axis of the femur, and ankle contact force was defined as the force acting on the talus from the tibia along the inferior-superior axis of the talus. Peak values were extracted from the first and second 50% of the stance phase for the baseline and retention trials. We report values for the eight subjects who retained a reduction in gastrocnemius activity during the retention trial. After ensuring normality using a Shapiro Wilk test, two-sided, paired t-tests were used to compare the first and second peaks of the hip and ankle contact forces between trials. Due to the exploratory nature of this analysis, p-values were not corrected for multiple comparisons. All values are reported as mean ± standard deviation, and $\alpha=0.05$.

There were not significant changes in the first (0.13±0.22 BW, 3.3±5.3%, $p=0.142$, t-test, $n=8$) or second peak (-0.17±0.49 BW, -4.1±11%, $p=0.358$, t-test, $n=8$) of hip contact force between the baseline and retention trials (Fig. S2). There were also not significant changes in the first (0.38±0.57 BW, 13±19%, $p=0.098$, t-test, $n=8$) or second peak (-0.23±0.36 BW, -4.4±6.9%, $p=0.122$, t-test, $n=8$) of ankle contact force between the baseline and retention trials. However, some individuals experienced increased contact force (Fig. S2), especially during early stance at the ankle. Interestingly, despite the biofeedback design simulation predicting increased co-activation between the hamstrings and hip flexors (Fig. 3), which would increase hip contact force, we did not observe an increase in hip contact force. This aligns with our electromyography findings—there were no increases in late-stance hamstrings muscle activity during the retention trial (Fig. 5).

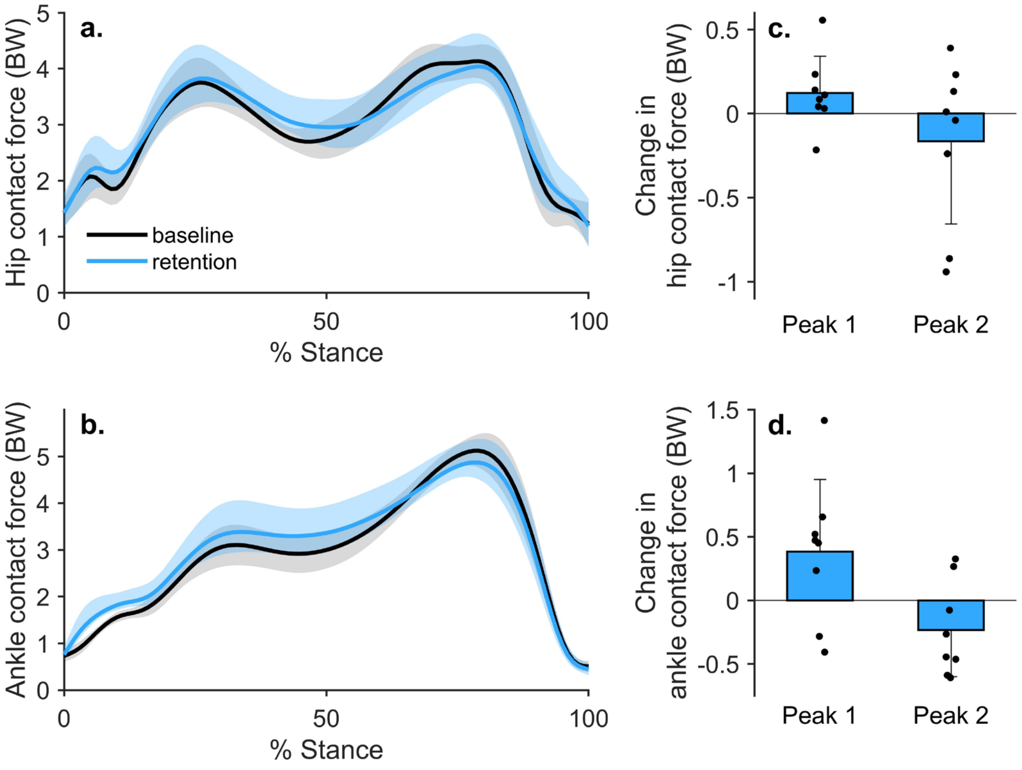

Figure S2: The effect of gastrocnemius avoidance gait on hip and ankle contact forces. (a, b) The mean (line) and standard deviation (shading) of hip and ankle contact force, expressed in bodyweights (BW), during the baseline and retention trials for the eight individuals who walked with reduced gastrocnemius activation during the retention trial. **(c, d)** The mean (bar), standard deviation (error bar), and individual (dot) changes in the first and second peak hip and ankle contact forces. There were no significant group differences in peak contact forces at either joint (p=0.098–0.358, paired t-tests, n=8), however some participants walked with increased contact forces as large as 1.4 BW.

Effect of gastrocnemius avoidance gait on the contralateral limb

Participants were given visual electromyography biofeedback on a single limb and were instructed to walk without changing their kinematics. Figure 7 shows that they did not retain baseline walking knee kinetics on the feedback limb, and it is important to evaluate if their kinematics or kinetics changed on the non-feedback limb (i.e., the contralateral limb).

We averaged sagittal-plane, lower-extremity kinematics and kinetics over the final 30 contralateral limb stance phases for the baseline and retention (gastrocnemius avoidance gait) trials for the eight participants who retained a reduction in gastrocnemius activation during the retention trial. Early (0-50% stance) and late-stance (50-100% stance) peaks were extracted from the hip and knee curves, and a late-stance peak was extracted from the ankle curves, as described in the Methods. We tested for normality using a Shapiro Wilk test. Normally distributed data from the baseline and retention trials were compared using two-sided, paired t-tests, and non-normally distributed data were compared using Wilcoxon signed rank tests. Due to the exploratory nature of this analysis, p-values were not corrected for multiple comparisons. All values are reported as mean ± standard deviation, and $\alpha=0.05$.

From the baseline to the retention trial, there were no significant differences in the early or late-stance peaks of the hip flexion angle ($p=0.493$, $p=0.551$, respectively; t-tests; $n=8$) or moment ($p=0.738$, $p=0.078$, respectively; t-test, Wilcoxon signed rank test, respectively; $n=8$, Fig. S3). Compared to baseline, the early-stance knee flexion angle was 2.7±2.8° (14±15%, $p=0.039$, Wilcoxon signed rank test, $n=8$) greater during the retention trial, but the late-stance minimum knee flexion angle was not significantly different between trials ($p=0.083$, t-test, $n=8$). Compared to baseline, the early-stance knee extension moment was 0.94±0.93 %BW*ht (35±36%, $p=0.024$, t-test, $n=8$) greater during the retention trial, and the late-stance flexion moment peak was 0.72±0.62 %BW*ht (47±42%, $p=0.014$, t-test, $n=8$) smaller. There were no significant differences in the late-

stance peaks of the ankle plantarflexion angle ($p=0.292$, t-test, $n=8$) or moment ($p=0.165$, t-test, $n=8$) between the baseline and retention trials.

When walking with the gastrocnemius avoidance gait pattern, participants increased their early-stance knee extension moment in the contralateral limb, on average. An elevated knee extension moment has been related to accelerated medial compartment cartilage degeneration[7], likely because it increases early-stance knee contact force. This elevated loading would be undesirable, especially for participants with bilateral knee osteoarthritis. Future studies should investigate if these contralateral limb changes are mitigated by providing bilateral electromyography biofeedback or by a long-term retraining protocol. Alternatively, participants could be given verbal instruction or biofeedback that discourages an increase in the knee flexion angle or moment. Without mitigating this increase in knee flexion moment, unilateral gastrocnemius avoidance retraining should likely not be prescribed to individuals with knee osteoarthritis due to the potential for a harmful elevation of loading in their contralateral knee.

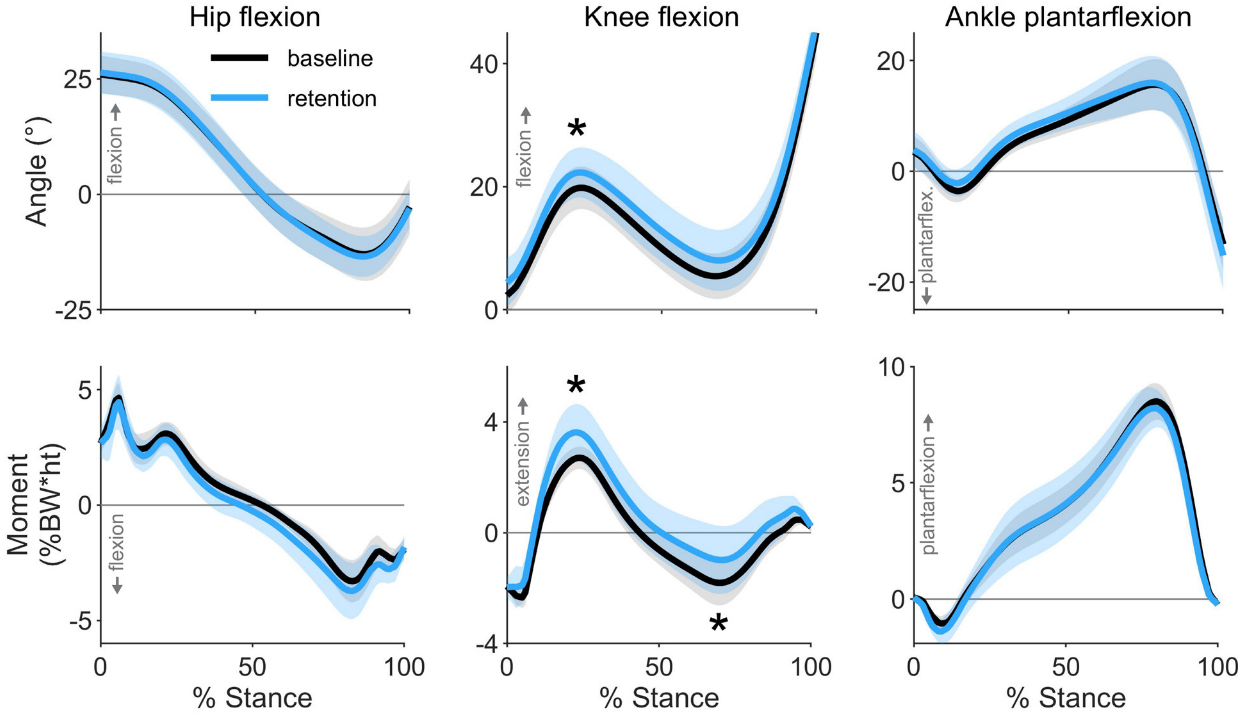

Figure S3: Kinematics and kinetics of the contralateral limb. The mean (line) and standard deviation (shading) of sagittal-plane joint angles (top) and moments (bottom) for the limb that did not receive real-time visual electromyography biofeedback (*$p<0.05$). The baseline (natural walking) and retention (gastrocnemius avoidance gait) trials are plotted for the eight subjects who walked with reduced gastrocnemius activation during the retention trial. There were no significant differences in peak hip or ankle kinematics or kinetics (p=0.078-0.738; paired t-tests and Wilcoxon signed rank test; n=8). The early-stance knee flexion angle and knee extension moment were greater than baseline (p=0.039, p=0.024, respectively; Wilcoxon signed rank test, paired t-test, respectively; n=8), and the late-stance knee flexion moment was smaller than baseline (p=0.014, paired t-test, n=8).

Explanation of biofeedback given to participants during the experiment

These instructions were read to all participants to explain the coordination retraining biofeedback.

1) Prior to standing calf raises:

> "The goal of this study is to teach you to change the amount that you activate two of your main calf muscles. The gastrocnemius crosses both the ankle and the knee and is higher up and more superficial on your lower leg. The soleus muscle only crosses the ankle joint, and is further down, and deeper in your lower leg [*show images of muscles and palpate them*]. We will teach you to reduce the ratio of your gastrocnemius activity to soleus activity, or put another way, to reduce your gastrocnemius activity and increase your soleus activity. The feedback you will be getting is a bar representing the gastrocnemius:soleus ratio. A high number is bad, a low number is good. Your goal will be to get the magnitude of this bar to be below the target line. We are going to practice with calf raises first. You will feel your gastrocnemius further up in your calf, and your soleus further down. When doing standing calf raises, you use more gastrocnemius than soleus. Do 10 calf raises and feel the activation of your gastrocnemius."

2) Prior to seated calf raises:

> "When you do seated calf raises, you activate your soleus more than your gastrocnemius. Do 10 seated calf raises and try to feel your soleus activating. You will want to feel yourself activating this muscle during walking more than your gastrocnemius which you felt during standing calf raises."

3) Prior to first feedback trial (bar magnitude only):

"During this trial, you are going to try to reduce your gastrocnemius:soleus ratio during walking. Feel free to explore different strategies but try to walk as normally as possible with regards to how your legs are moving (your kinematics). You should feel the activation in your soleus lower and deeper than your gastrocnemius. You will get 4 minutes to explore. For the fifth minute, we will have you walk consistently with your most successful strategy. For the final minute, you will continue walking this way, but you will not be getting feedback."

4) Prior to second feedback trial (bar magnitude and color):

"During this trial, you will be getting two types of feedback per step. The magnitude of the bar will continue to represent the ratio between your gastrocnemius and soleus activation. The bar will now be colored with a gradient that represents the magnitude of gastroc activation. Red coloring means high activation, which is bad, green means low activation, which is good, yellow means activation similar to baseline, which is ok. During the next two trials, continue trying to keep your bar below the line, while also making the color of the bar green. Similar to the last trial, these two trials will last for 6 minutes each. You can explore during the first four minutes, walk consistently with your best solution during the fifth minute, and try to retain it without feedback during the final minute."

5) Prior to the retention trial (no feedback):

"Continue walking with the most successful strategy that you learned from the feedback during the previous trials, but you will not receive feedback."

Hip abductor muscle path adjustments

We adjusted the origin and insertion points of the hip abductors in the model described by Rajagopal et al.[8] to improve estimates of hip flexor muscle activation (Fig. S4). During simulations of normal walking, static optimization was requiring non-physiologically large iliacus and psoas activations despite hip moments that matched normative values in the literature. The moment-generating capacity of the hip flexors was validated by Rajagopal et al.; however, this study investigated the sagittal plane moment in isolation, not in combination with frontal or transverse plane moments. We determined that the high hip flexor muscle activity may have, in part, resulted from the combined hip flexion and abduction moments that are generated during the latter half of stance phase of walking. When this model is in hip extension, all six of the gluteus medius and gluteus minimus muscle fibers, which generate the majority of the hip abduction moment, have sagittal plane moment arms that extend the hip (Fig. S5). During late stance, these muscles generate an antagonistic hip extension moment that requires elevated activation of the iliacus and psoas. Experimental studies[9,10], finite element models[11], and other MRI-based musculoskeletal models[12] suggest that the anterior fibers of the gluteus medius and minimus generate a hip flexion moment when the hip is in an extended position. We adjusted the origin and insertion points of the gluteus medius, gluteus minimus, and tensor fascia latae to match these data and models more closely (Fig. S5).

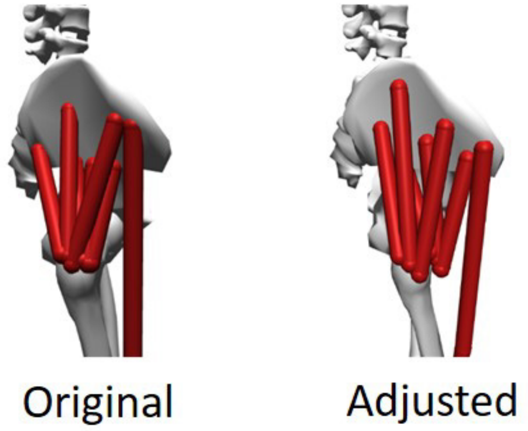

Figure S4: Changes in hip abductor muscle paths. We modified the origin and insertion points of the hip abductors from the original Rajagopal et al.[8] model (left) to the adjusted model (right). The origin of the gluteus medius and minimus were moved superiorly and laterally to increase the abduction moment arm to match experimental data. The insertion of the gluteus medius and minimus were moved anteriorly to allow them to generate a greater flexion moment to match experimental and model-based moment arms (Fig. S5).

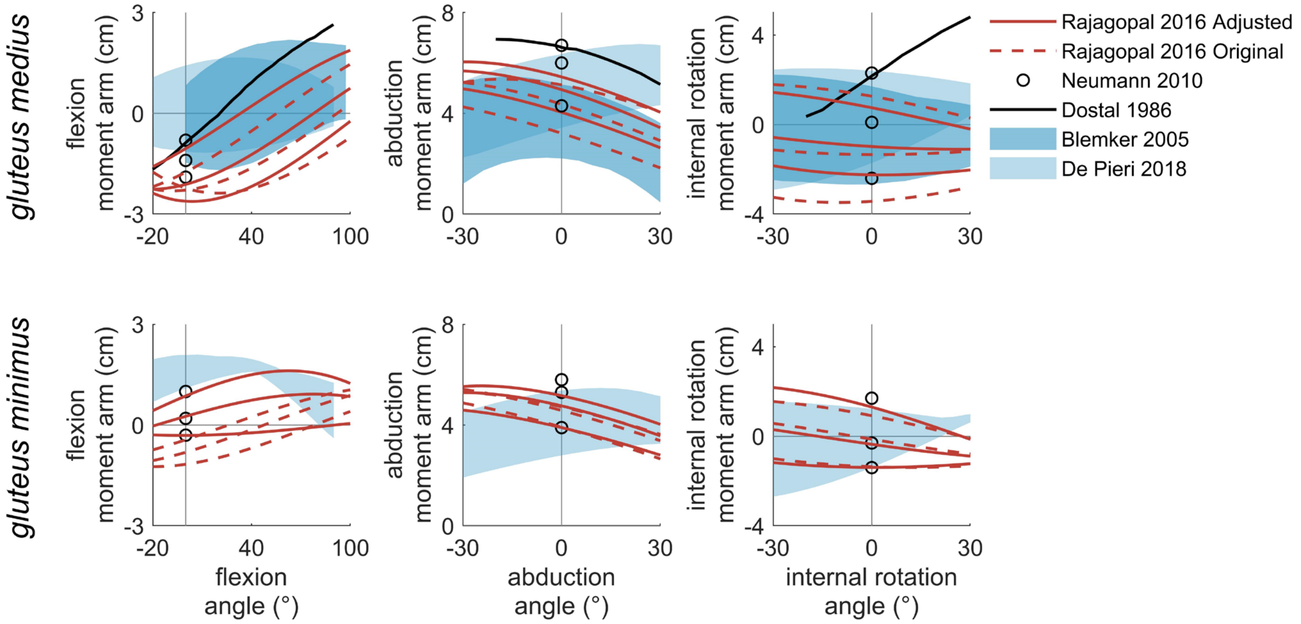

Figure S5: Hip abductor moment arms. The original and adjusted moment arms for the musculoskeletal model described in Rajagopal et al.[8] compared to moment arms from experiments (Dostal et al.[9], Neumann[10]), finite element models (Blemker et al.[11]), and MRI (De Pieri et al.[12]). The Dostal et al. moment arm curve is from the anterior region of the gluteus medius, and all other moment arms are from a range of fibers from the posterior to the anterior regions of the muscles.

Passive muscle force calibration

The muscles in the Rajagopal et al.[8] model generate passive joint moments that are larger than those that have been measured in experiments[13], especially at large knee or hip flexion angles[14] (Fig. S6). Previous studies have addressed this by changing the optimal muscle fiber lengths, tendon slack lengths, or muscle geometry[14]. These changes were driven by passive muscle forces but they also affect the active force-generating capacity of the muscle. Another approach to modifying the passive muscle forces is to modify the passive muscle force-length curve, which does not affect the active force-generating capacity of the muscle.

We calibrated the passive force-length curves for each muscle in the Rajagopal model to more closely match experimentally-measured passive joint moment curves[13]. Using constrained optimization in MATLAB, we minimized the root mean square difference between passive joint moment curves from the model and experiment. As design variables, we used two of the values that parameterize the passive force-length curve in the Millard et al.[15] muscle model: the muscle length (l^m) when it begins to generate force (default value: l_o^m) and the length at which passive muscle force reaches its optimal fiber force (F_o^m, default value: 1.7 l_o^m). We limited changes in l^m values to 0.2 l_o^m above or below their default values and added an L2 regularization term to the objective function to penalize large deviations in l^m from default values. The resulting changes in passive joint moments are shown in Figure S6, and changes in the l^m values that parameterize the passive force-length curve are shown in Figure S7 and Table S1. After calibration, the quadriceps and gluteus maximus muscles all began generating passive muscle forces at longer muscle fiber lengths, which reduces the passive moments generated at large knee and hip flexion moments. The iliacus and psoas began generating passive force at shorter muscle fiber lengths, which allows them to generate a larger passive hip flexion moment when the hip is extended. Incorporating calibrated passive muscle forces into our static optimization tool helped reduce large hip flexor muscle activations during

stance phase as well as quadriceps-hamstrings co-contraction when the knee is flexed during swing phase.

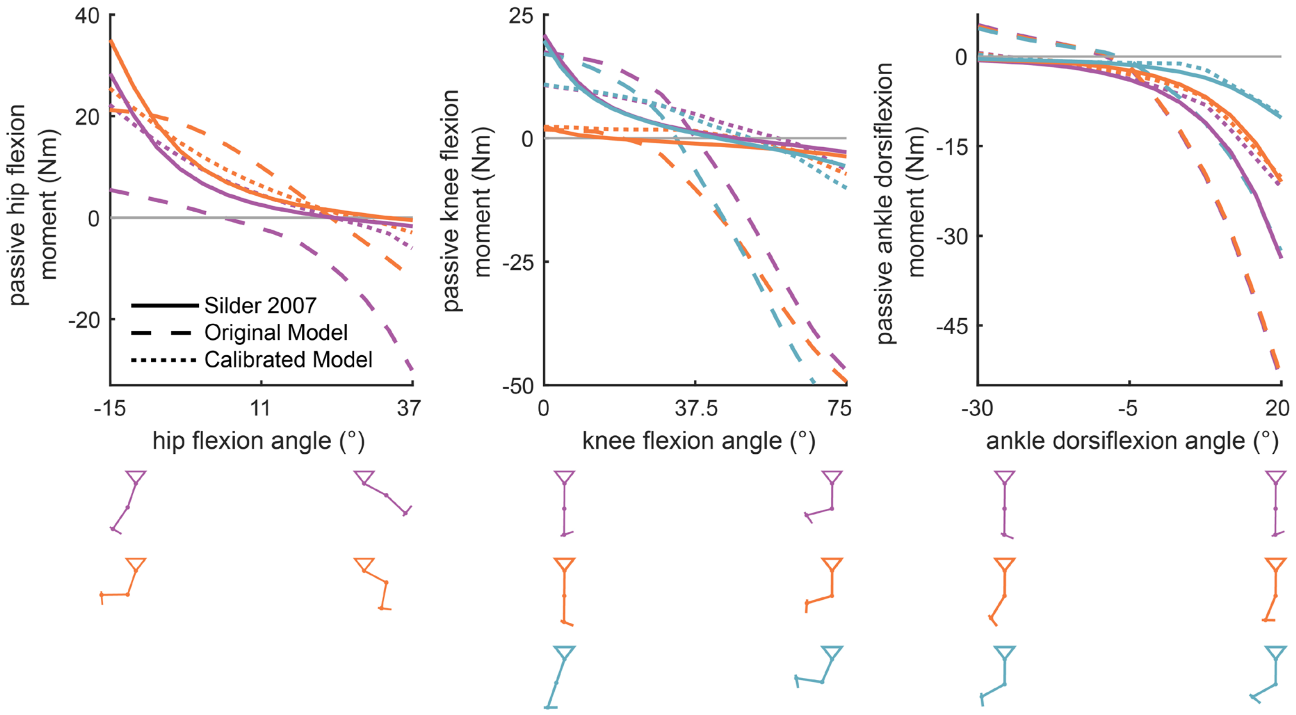

Figure S6: Sagittal plane passive joint moment curves for the hip, knee, and ankle. Calibration improved the agreement in passive joint moments between the Rajagopal et al.[8] model and experimentally-measured moments from Silder et al.[13]. Each joint was moved over the shown range of motion with other joints fixed at various angles (see Silder et al.). For example, the hip was moved from 15° of extension to 37° of flexion (left) with the knee fixed at 15° (purple) and 60° (orange).

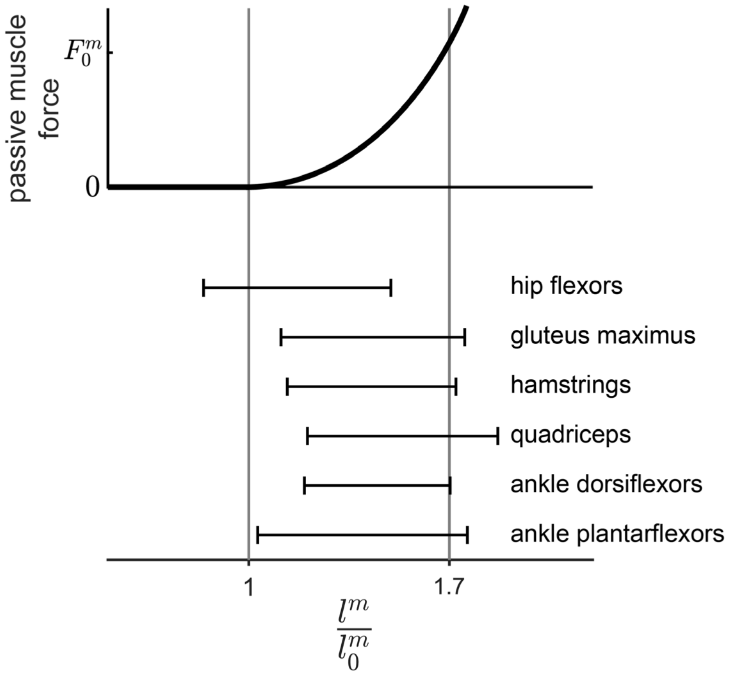

Figure S7: Calibrated normalized muscle fiber lengths that parameterize the passive muscle force curve for sagittal plane muscle groups. At $\boldsymbol{F^m = 0}$, the default $\boldsymbol{l^m/l_o^m = 1}$. At $\boldsymbol{F^m = F_o^m}$, the default $\boldsymbol{l^m/l_o^m = 1.7}$. Muscle groupings are defined in Table S1. With the exception of the hip flexors, most muscle groups begin generating passive force at a longer fiber length after calibration.

Table S1: Calibrated passive muscle force curve parameters for the Rajagopal et al.[8] model. Muscle name abbreviations correspond to the muscle names in the model.

Muscle name	Muscle name abbreviation	$\frac{l^m}{l_o^m}$ at $F^m = 0$	$\frac{l^m}{l_o^m}$ at $F^m = F_o^m$
**default value**		1	1.70
hip flexors			
psoas	psoas	0.93	1.50
iliacus	iliacus	0.88	1.50
adductor longus	addlong	0.82	1.50
tensor fascia latae	tfl	1.20	1.72
gluteus maximus			
gluteus maximus (superior)	glmax1	0.92	1.84
gluteus maximus (middle)	glmax2	1.20	1.72
gluteus maximus (inferior)	glmax3	1.20	1.71
hamstrings			
biceps femoris long head	bflh	1.20	1.71
biceps femoris short head	bfsh	1.14	1.70
semimembranosus	semimem	1.20	1.75
semitendinosus	semiten	1.18	1.71
gracilis	grac	0.80	1.66
sartorius	sart	0.80	1.50
quadriceps			
vastus medialis	vasmed	1.20	1.90
vastus lateralis	vaslat	1.20	1.90
vastus intermedius	vasint	1.20	1.90
rectus femoris	recfem	1.20	1.90
ankle dorsiflexors			
tibialis anterior	tibant	1.19	1.71
ankle plantarflexors			
gastrocnemius medialis	gasmed	1.20	1.90
gastrocnemius lateralis	gaslat	1.20	1.67
soleus	soleus	1.20	1.90
tibialis posterior	tibost	0.80	1.50
other			
adductor brevis	addbrev	0.96	1.67
adductor magnus (distal)	addmagDist	1.00	1.70
adductor magnus (ischial)	addmagIsch	1.01	1.70
adductor magnus (middle)	addmagMid	1.00	1.70
adductor magnus (proximal)	addmagProx	1.00	1.70
gluteus medius (anterior)	glmed1	1.00	1.70
gluteus medius (middle)	glmed2	1.06	1.70
gluteus medius (posterior)	glmed3	1.12	1.70
gluteus minimus (anterior)	glmin1	1.00	1.70
gluteus minimus (middle)	glmin2	1.00	1.70
gluteus minimus (posterior)	glmin3	1.01	1.70
piriformis	piri	1.11	1.70
peroneus brevis	perbrev	0.92	1.69
peroneus longus	perlong	0.80	1.50
extensor digitorum longus	edl	1.12	1.72
extensor hallucis longus	ehl	1.09	1.73
flexor digitorum longus	fdl	0.95	1.70
flexor hallucis longus	fhl	1.04	1.70

Tendon compliance sensitivity analysis

Our static optimization implementation estimates the effect of tendon compliance by computing the muscle length that yields static equilibrium between the muscle and tendon, using the activation from the previous step. However, due to the implementation of the muscle model[15] in OpenSim, the effects of muscle velocity are ignored when computing muscle-tendon force static equilibrium. Thus, there is a tradeoff between two muscle modeling approaches: 1) a rigid tendon and muscle force-velocity effect and 2) a compliant tendon and no muscle force-velocity effect. To select which approach to use in our simulations, we simulated a single gait cycle of natural walking from the baseline trial for all 10 participants without tracking electromyography. We selected the modeling approach that minimized the sum of the mean absolute error between simulated activation and measured electromyography, averaged across the muscles of greatest interest for this study: the gastrocnemius medialis, gastrocnemius lateralis, and soleus.

The mean absolute error in activation, averaged across muscles, was 0.07 for the rigid tendon approach, and 0.05 for the compliant tendon approach (Fig. S8). The differences between the rigid and compliant tendon were small for both gastrocnemii; however, the compliant tendon approach resulted in soleus activations that more closely matched electromyography, compared to the rigid tendon approach.

This analysis led us to use the compliant tendon approach for all simulations in this study. This finding aligns with a previous dynamic simulation study showing that when tendon compliance is modeled, the ankle plantarflexors generate force at small velocities during the stance phase[16], further supporting our selection to model tendon compliance and not the force-velocity effect in this study. We evaluated the error for the three most important muscles in this study during a single activity; these conclusions should not be extrapolated to other muscles or activities without further testing. General conclusions about the importance of tendon compliance or the muscle force-velocity property of muscle should also not be drawn from this analysis.

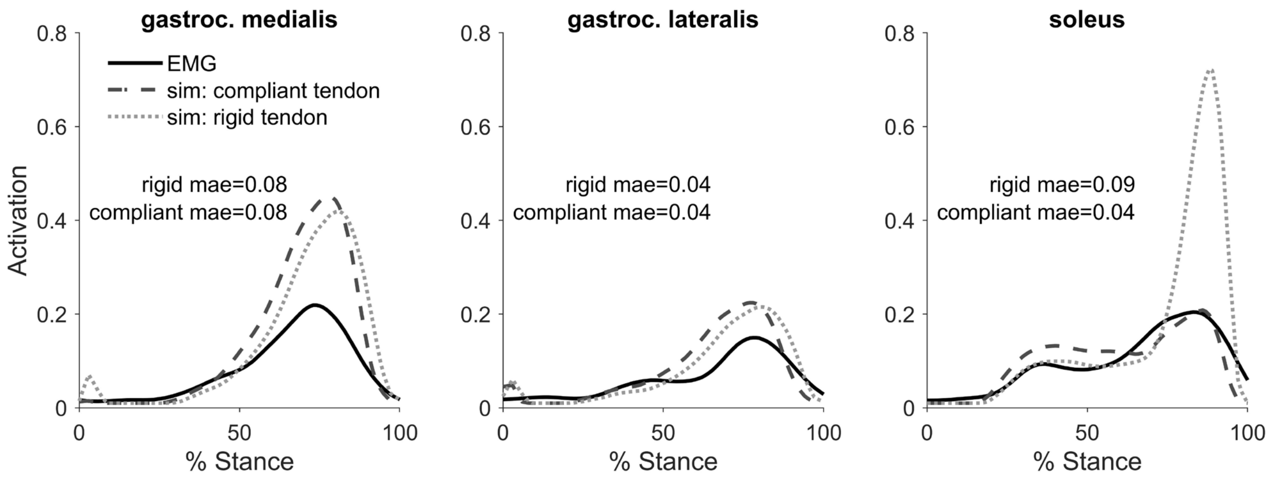

Figure S8: Sensitivity of simulated muscle activations to tendon compliance. Electromyography (EMG) and simulated (sim) muscle activations averaged across all 10 participants walking naturally. The rigid tendon simulations used muscles with a rigid tendon but incorporated the force-velocity property of muscle. The compliant tendon simulations estimated tendon compliance but ignored the force-velocity property of muscle. The mean absolute error (mae) averaged across all three muscles was 0.07 for the rigid tendon approach and 0.05 for the compliant tendon approach. Due to the lower error, the compliant tendon approach was used for all other simulations in this study.

References

1. Hurwitz, D. E., Ryals, A. B., Case, J. P., Block, J. A. & Andriacchi, T. P. The knee adduction moment during gait in subjects with knee osteoarthritis is more closely correlated with static alignment than radiographic disease severity, toe out angle and pain. *J. Orthop. Res.* **20**, 101–107 (2002).

2. Sharma, L. *et al.* Knee adduction moment, serum hyaluronan level, and disease severity in medial tibiofemoral osteoarthritis. *Arthritis Rheum.* **41**, 1233–1240 (1998).

3. Miyazaki, T. *et al.* Dynamic load at baseline can predict radiographic disease progression in medial compartment knee osteoarthritis. *Ann. Rheum. Dis.* **61**, 617–622 (2002).

4. Brisson, N. M., Gatti, A. A., Damm, P., Duda, G. N. & Maly, M. R. Association of Machine Learning-Based Predictions of Medial Knee Contact Force With Cartilage Loss Over 2.5 Years in Knee Osteoarthritis. *Arthritis Rheumatol.* **73**, 1638–1645 (2021).

5. Winby, C. R., Lloyd, D. G., Besier, T. F. & Kirk, T. B. Muscle and external load contribution to knee joint contact loads during normal gait. *J. Biomech.* **42**, 2294–2300 (2009).

6. Walter, J. P., D'Lima, D. D., Colwell, C. W. & Fregly, B. J. Decreased knee adduction moment does not guarantee decreased medial contact force during gait. *J. Orthop. Res.* **28**, 1348–1354 (2010).

7. Chehab, E. F., Favre, J., Erhart-Hledik, J. C. & Andriacchi, T. P. Baseline knee adduction and flexion moments during walking are both associated with 5 year cartilage changes in patients with medial knee osteoarthritis. *Osteoarthr. Cartil.* **22**, 1833–1839 (2014).

8. Rajagopal, A. *et al.* Full-Body Musculoskeletal Model for Muscle-Driven Simulation of Human Gait. *IEEE Trans. Biomed. Eng.* **63**, 2068–2079 (2016).

9. Dostal, W. F., Soderberg, G. L. & Andrews, J. G. Actions of Hip Muscles. *Phys. Ther.* **66**, 351–359 (1986).

10. Neumann, D. A. Kinesiology of the hip: A focus on muscular actions. *J. Orthop. Sports Phys. Ther.* **40**, 82–94 (2010).

11. Blemker, S. S. & Delp, S. L. Three-dimensional representation of complex muscle architectures and geometries. *Ann. Biomed. Eng.* **33**, 661–673 (2005).

12. De Pieri, E. *et al.* Refining muscle geometry and wrapping in the TLEM 2 model for improved hip contact force prediction. *PLoS One* **13**, https://doi.org/10.1371/journal.pone.0204109 (2018).

13. Silder, A., Whittington, B., Heiderscheit, B. & Thelen, D. G. Identification of passive elastic joint moment-angle relationships in the lower extremity. *J. Biomech.* **40**, 2628–2635 (2007).

14. Lai, A. K. M., Arnold, A. S. & Wakeling, J. M. Why are Antagonist Muscles Co-activated in My Simulation? A Musculoskeletal Model for Analysing Human Locomotor Tasks. *Ann. Biomed. Eng.* **45**, 2762–2774 (2017).

15. Millard, M., Uchida, T., Seth, A. & Delp, S. L. Flexing computational muscle: Modeling and simulation of musculotendon dynamics. *J. Biomech. Eng.* **135**, 021005 (2013).

16. Arnold, E. M., Hamner, S. R., Seth, A., Millard, M. & Delp, S. L. How muscle fiber lengths and velocities affect muscle force generation as humans walk and run at different speeds. *J. Exp. Biol.* **216**, 2150–2160 (2013).
